# A modified Goldstein filter for interferogram denoising of interferometric imaging radar altimeter based on multiple quality-guided graphs

**DOI:** 10.1371/journal.pone.0308636

**Published:** 2024-08-08

**Authors:** Jian Liu, Huili Zhang, Lihua Wang, Zhiyong Wang

**Affiliations:** 1 Nanjing Center, China Geological Survey, Nanjing, Jiangsu Province, China; 2 Spatial Information Technology Application Department, Changiiang River Scientific Research Institute, Wuhan, Hubei Province, China; 3 College of Geodesy and Geomatics, Shandong University of Science and Technology, Qingdao, Shandong Province, China; ICIMOD: International Centre for Integrated Mountain Development, NEPAL

## Abstract

Aiming at the characteristics that the signal noise ratio (SNR) gradually decreases from the near to far range of the swath, an adaptive phase filtering algorithm based on Goldstein filtering and combined with multiple quality-guided graphs was proposed. Firstly, the components used to determine the filtering parameters were obtained through residue density, pseudo-coherence coefficient and pseudo-SNR, the three quality-guided graphs. Then, the filter parameters were calculated by weighting the three components. Finally, the size of filtering window was determined according to the account of residues, and the interferometric phase noise was removed in frequency domain. Simulated data, TSX/TDX data and airborne interferometric imaging radar altimeter data were used to verify the performance of the new algorithm. Compared with the results of Goldstein filtering and its improved algorithms, the results showed that the proposed algorithm can effectively filter out phase noise while maintaining the edge characteristics of interferometric fringe. The section of filtering result can well match with the section of simulated pure interfeometric phase. Moreover, the algorithm proposed in this paper can effectively remove the noise in the interferogram of TSX/TDX sea ice data, and the residues’ filtering rate was above 86%, which can effectively remove the phase residues of the sea ice surface while maintaining the characteristics of the sea ice edge. Experimental results showed that the new algorithm provides an effective phase noise filtering method for imaging radar altimeter data processing.

## Introduction

As a new type of radar altimeter, Interferometric imaging radar altimeter (InIRA) integrates the near nadir observation, height tracking measurement, synthetic aperture and interferometric technology, etc., and it has the characteristics of wide swath, high resolution and 3D imaging [1–3]. Conceived as a major new tool for climate studies, the Surface Water and Ocean Topography (SWOT) [4–7] satellite mission launched December 16th 2022. On September 15, 2016, the Chinese Tiangong-2 space laboratory with an onboard InIRA [8–10] was launched. Several research results show that InIRA has a good application prospect in sea surface height, wind speed inversion, sea target recognition and so on [11–14].

**Fig 1:**
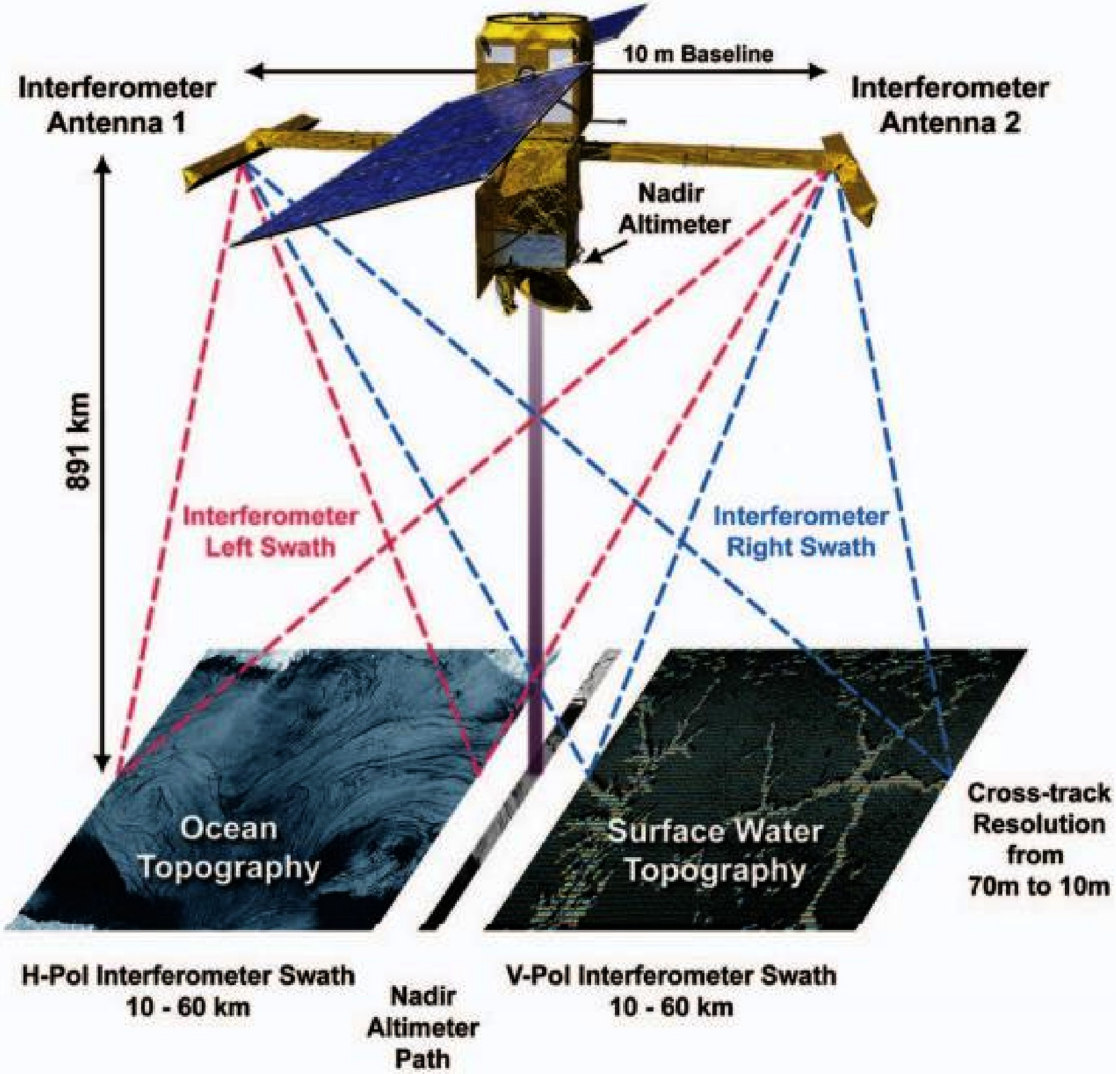
The working diagram of InIRA.

As shown in Fig 1, the most distinctive feature of the imaging radar altimeter is that the near-nadir observation with a small incidence angle, ranging from 1° to 8° [8, 15]. The working mechanism of small incidence angle makes the imaging radar altimeter data differ greatly in the near and far range of image, mainly including resolution, backscattering, echo intensity, etc.[6, 7, 16, 17]. With the increase of the incidence angle, the backscatter intensity becomes weaker. At the remote part of the interferogram, the image SNR and the information contained in the echo signal decreases, and the noise increases [9, 18]. In addition, the InIRA uses dual antennas for detection, so that the temporal baseline of interferometry is zero, that is, the noise caused by time decoherence can be ignored [19, 20].

Besides temporal, there can be phase noise from thermal or geometrical sources, Doppler centroids, processing-induced decorrelation and other sources [21]. Interferometric phases need to be filtered before further processing and application. Algorithms such as Goldstein filter and its improved filters have been widely used in radar interferometric processing [22–29], but traditional phase filtering algorithms cannot be effectively applied in imaging radar altimeter interferometry. Small incidence angle and near nadir observation mode of imaging radar altimeter makes some difference between the near and far range of image. Goldstein filter and its improved filtering algorithms rely on the global unification or a single quality graph to determine the filter parameters, which cannot accurately reflect the noise distribution from near and far range of imaging radar altimeter image. Xiao Dong et al. [30] proposed a non-uniform phase filtering algorithm by establishing the relationship between multi-look number and measurement accuracy. The filtering window is chosen according to the relationship among the height variation, coherence, and the imaging geometry. The algorithm is effective for ocean topography measurement. But, the algorithm needs the expected accuracy to calculate the number of the multi-look processing, so it has some limitations. Lu Xiang et al. [31] proposed a total variation regularization filtering which based on the features of noise distribution along the cross-track direction for the altimeter. It ensures the consistency of sea surface height inversion accuracy and reduces the loss of the phase information of small sea surface targets. The varying random noise of flattened phase can be attenuated effectively with the algorithm. However, the selection of its parameters lacks some objectivity, and there are some limitations in maintaining the edge of the image. In view of the above problems, according to the characteristics of phase noise distribution in imaging radar altimeter images, an adaptive phase filtering algorithm based on Goldstein filtering algorithm and combined with multiple quality-guided graphs was proposed in this paper.

The structure of this paper is as follows, including four sections. The second section introduced the principle of different quality-guided graphs and new filtering algorithms. The third section introduced the results and discussion of the filtering algorithms, using simulated data, TanDEM/ TerraSAR-X (TSX/TDX) data and airborne InIRA data respectively. It also made some comparisons to the filtering results of different algorithms, and provided the quantitative comparison and evaluation. The fourth section summarized the research of this paper and displayed the specific conclusions obtained from the research.

## Materials and methods

According to the characteristics of the noise distribution in the imaging radar altimeter image and the bases of Goldstein filter, the filtering parameter was determined by multiple quality-guided maps, which can better reflect the local noise characteristics accurately. The filtering strength was controlled by noise degree adaptively. So, the phase noise can be effectively filtered out at the near range and far range of interferogram.

### Goldstein filtering

In 1998, Goldstein R.M et al. [24] proposed an interferometric phase filtering algorithm in frequency domain: Goldstein filtering. In this algorithm, the original interferogram is processed by two-dimensional Fourier transform, and then the power spectrum was smoothed in the frequency domain. Finally, the filtered interferogram is obtained by inverse Fourier transform. The specific algorithm steps are as follows:

The interferometric phase or interferogram is segmented into overlapping rectangular patches. Patches are overlapped to attenuate discontinuities at the boundaries, and the overlap degree is generally more than 75% [24]. Generally, the size of the patch is 32*32 pixels [26, 28, 32, 33].

Taking the two-dimensional Fourier transform to each image block *P(i*,*j)*:

B(u,v)=FFT2(P(i,j)) (1)

Filtering the power spectrum *B(u*,*v)*:

$$H(u,v)=S{\left\{\left|B(u,v)\right|\right\}}^{\alpha }\cdot B(u,v)$$ (2)

In Formula (2), *S*{} is the smoothing operator and *α* is the filtering parameter in the frequency domain. The filter parameter *α* is chosen between zero and one. When *α* = 0, the filter has no effect, while when *α* = 1, the strongest filtering is applied [26, 27]. *H*(*u*,*v*) is the smoothed power spectrum.

The inverse Fourier transform of *H*(*u*,*v*) is carried out to obtain the filtered interferogram *B*^’^(*u*,*v*):

$$B^{'}(u,v)=IFFT2(H(u,v))$$ (3)

### Multiple quality-guided graphs

#### Residues density

The *R*_*density*_ is the amount of the residues [29, 34] in each window, and the filter parameter component *alpha1* in the frequency domain was determined by *R*_*density*_. In an interferogram, the region with much noise and dense residues was strongly filtered, and the region with little noise and sparse residues was weakly filtered. The *alpha1* can be obtained through normalization:

$$alpha1=\frac{R_{density}}{Max(R_{density})}$$ (4)

**Fig 2:**
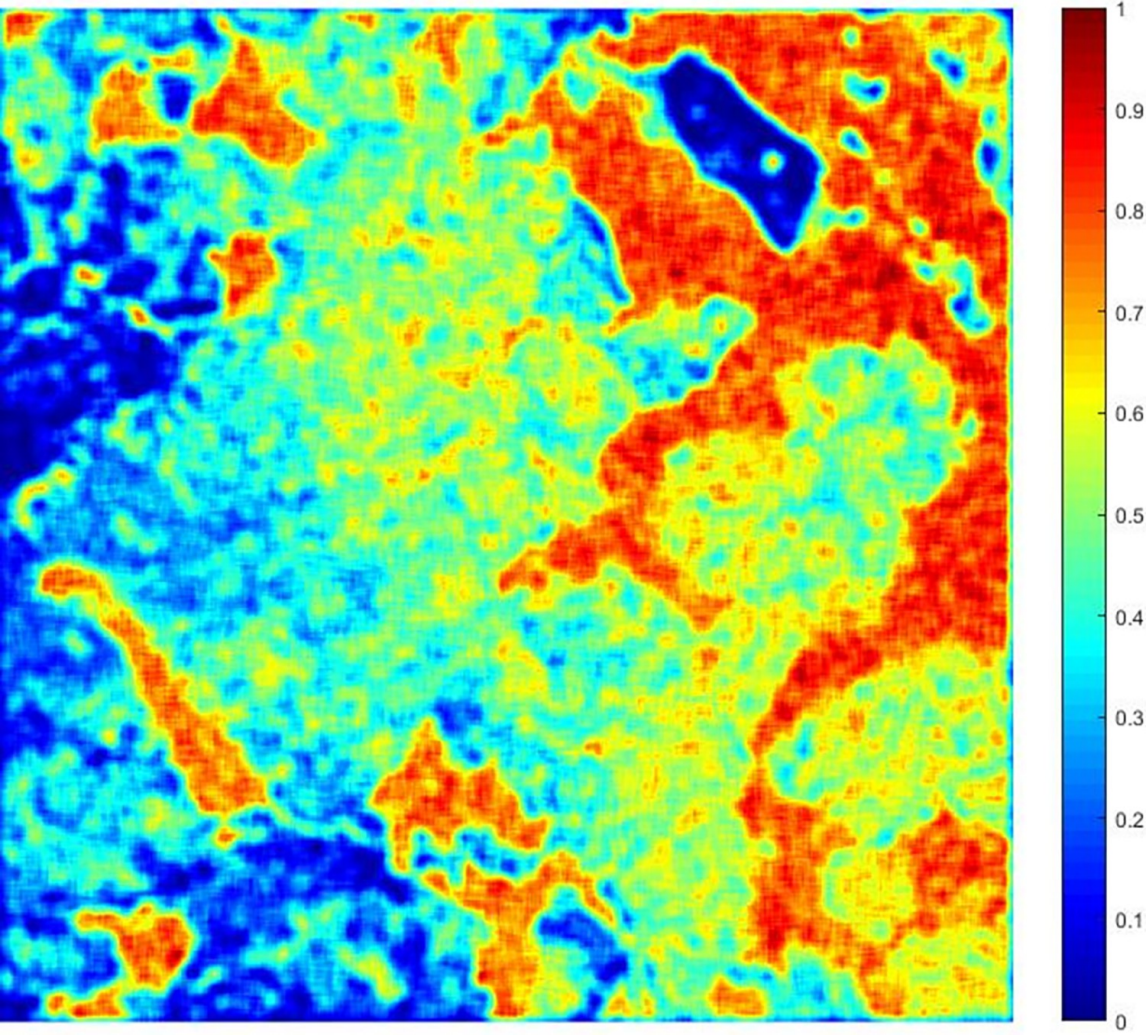
The normalized residues density graph of the TSX/TDX interferogram.

In Formula (4), *R*_*density*_ is the residues density of the filtering window, *Max(R*_*density*_*)* represents the maximum residues density of the whole image. The normalized residues density graph of the TSX/TDX interferogram is shown in Fig 2.

#### Pseudo-coherence coefficient

By calculating the *PC*, local pseudo-coherence coefficient of the interferogram, the filter parameters in the frequency domain were determined [26]. So, the regions with small *PC* and much noise were strongly filtered. The regions with large *PC* and small noise were weakly filtered. The *PC* can be obtained based on local statistics of the interferometric phase itself, which can be calculated by the formula:

$$PC=\frac{\left|\sum_{i=1}^{N}\varphi_{i}\right|}{\sum_{i=1}^{N}\left|\varphi_{i}\right|}$$ (5)

In Formula (5), *N* is the size of the sliding window used to calculate the pseudo-coherent graph, and φ is the complex phase value of the interferogram.

The filter parameter component *alpha2* was determined by *PC* of the interferometric phase:

$$alpha2=1-\bar{pc}$$ (6)

**Fig 3:**
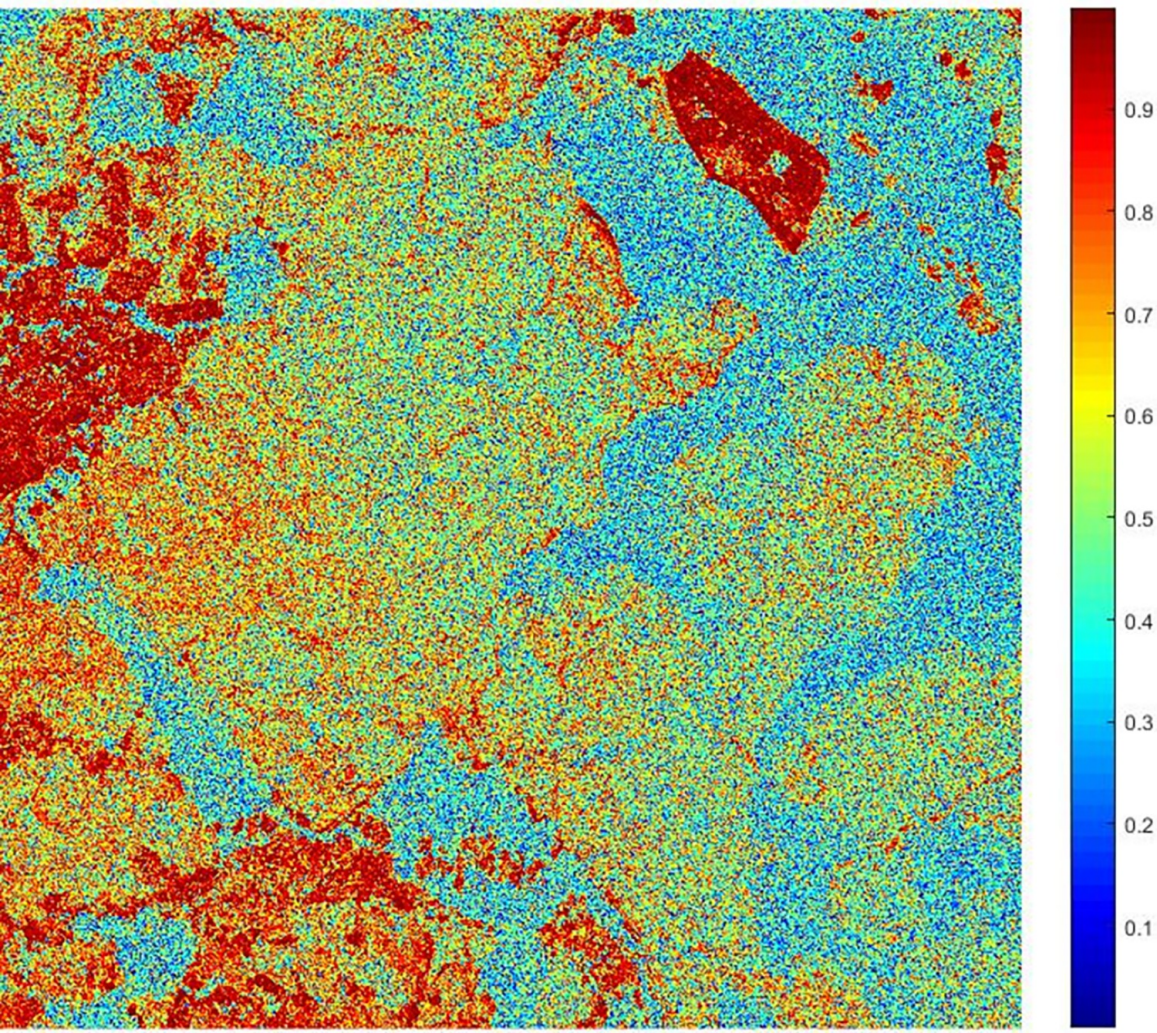
The pseudo-coherence coefficient graph of the TSX/TDX interferogram.

In Formula (6), $\bar{pc}$ is the mean value of *PC* of interferometric phase in filtering window. The pseudo-coherence coefficient graph of the TSX/TDX interferogram is shown in Fig 3.

### Pseudo-SNR

Pseudo-SNR (signal to noise ratio) is calculated by using the variance of interferometric phase value [28–35], that is, the phase variance ratio of signal and noise is used to determine the SNR of interferogram. Firstly, the $\delta_{\varphi }^{2}$ [24], local phase value variance of all pixels in the interferogram, is calculated by Formula (7). The maximum value of the local phase variance is taken as the signal variance, and the minimum value of the local phase variance is taken as the noise variance.


$$\delta_{\varphi }^{2}=\frac{\sum_{N}{\left(\varphi_{\mathrm{i,j}}-\bar{\varphi_{i,j}}\right)}^{2}}{N-1}$$ (7)

In Formula (7), *N* is the size of the local window and $\bar{\varphi_{i,j}}$ is the local linear phase gradient in the window, which can be obtained by the first-order difference of *φ*_*i*,*j*_ in the radar range direction and azimuth direction and calculating the mean value.

For local adaptive phase filtering, the *P*_*SNR*_ (Pseudo-SNR) can be obtained by taking the logarithm of the ratio between the maximum phase variance of the entire image ($\delta_{\varphi ,Max}^{2}$) and the phase value variance of the filtering window ($\delta_{\varphi ,Patch}^{2}$), which can be calculated by Formula (8):

$$P_{SNR}=10\log_{10}(\frac{\delta_{\varphi ,Max}^{2}}{\delta_{\varphi ,Patch}^{2}})$$ (8)

Normalizing the *P*_*SNR*_, the filter parameter component *alpha3* was obtained:

$$alpha3=1-(\frac{P_{SNR}}{Max(P_{SNR})})$$ (9)

**Fig 4:**
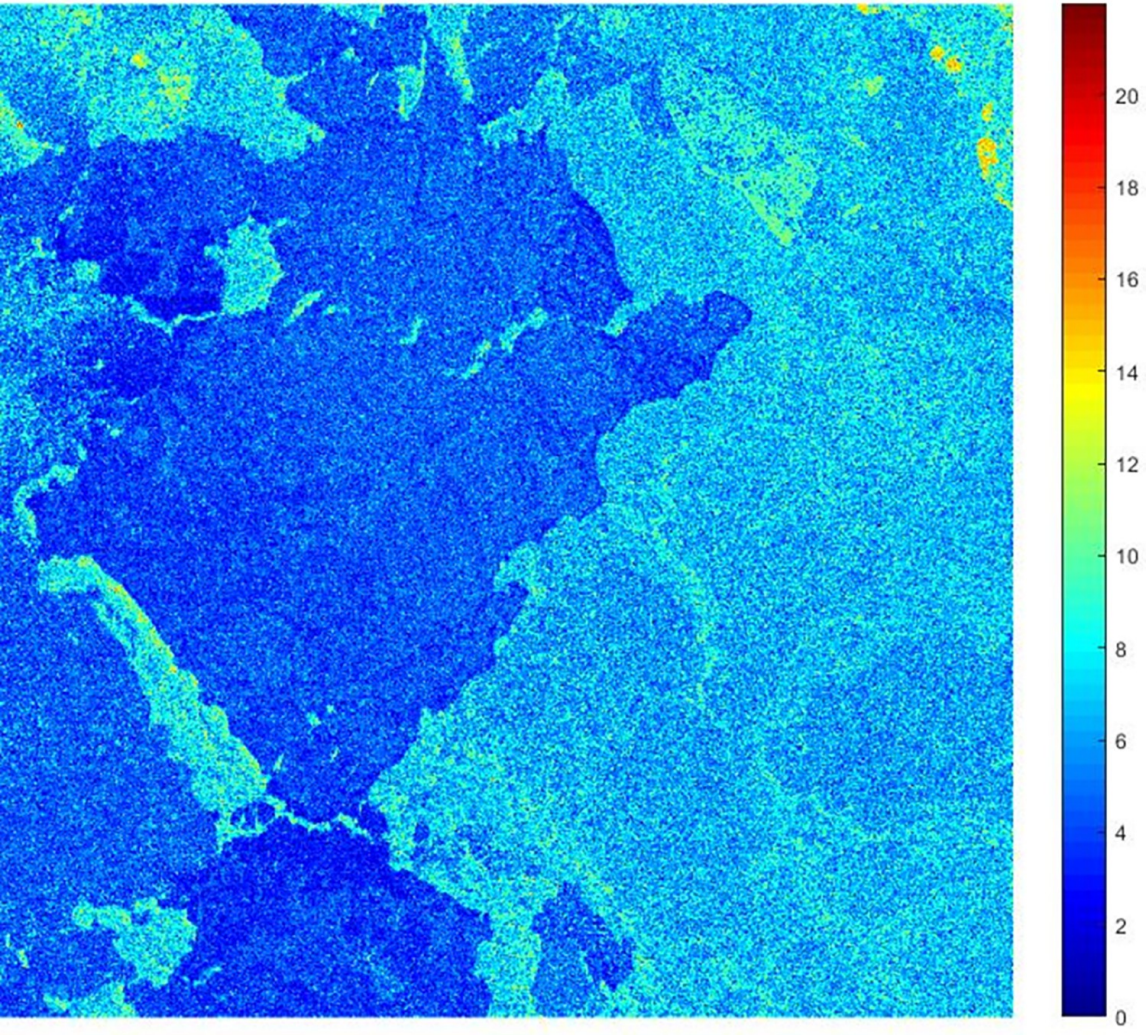
The pseudo-SNR graph of the TSX/TDX interferogram.

The pseudo-SNR graph of the TSX/TDX interferogram is shown in Fig 4.

### New filtering model

#### Filtering parameter

After the filter parameter components (*alpha1*, *alpha2*, *alpha3*) were obtained, the filter parameter *α* was calculated by weighting. To better remove phase residues, the principle of weight distribution is that the larger the filter parameter component is, the greater the weight it takes in the calculation of *α*. It can be obtained from Eq (10):

$$\alpha =\frac{1}{2}\alpha_{1}+\frac{1}{3}\alpha_{2}+\frac{1}{6}\alpha_{3}$$ (10)

In Formula (10), *α*_*1*_, *α*_*2*_ and *α*_*3*_ were the values assigned to components in decreasing order, i.e. α_1_ >α_2_ >α_3_.

#### Algorithm steps

Step1. Calculate the *R*_*density*_, *PC* and *P*_*SNR*_ of interferogram.

Step2. Use 64*64 sliding window to crop the image, step size is 4, overlap degree is 28.

Step3. Calculate the filter parameter components *alpha1*, *alpha2* and *alpha3*.

Step4. Calculate the frequency domain filtering parameter *α*.

Step5. Smooth processing in frequency domain. Use the mean value of 5*5 to convolve the window to smooth the image and filter out the noise.

Step6. Perform iterative processing. In order to further remove the phase residual error, the first filtering result was returned to Step1 for iterative operation, and the filtering window of 32*32 was adopted for the second filtering, and the filtering parameters were recalculated.

Step7. Acquire the filtered interferogram.

**Fig 5:**
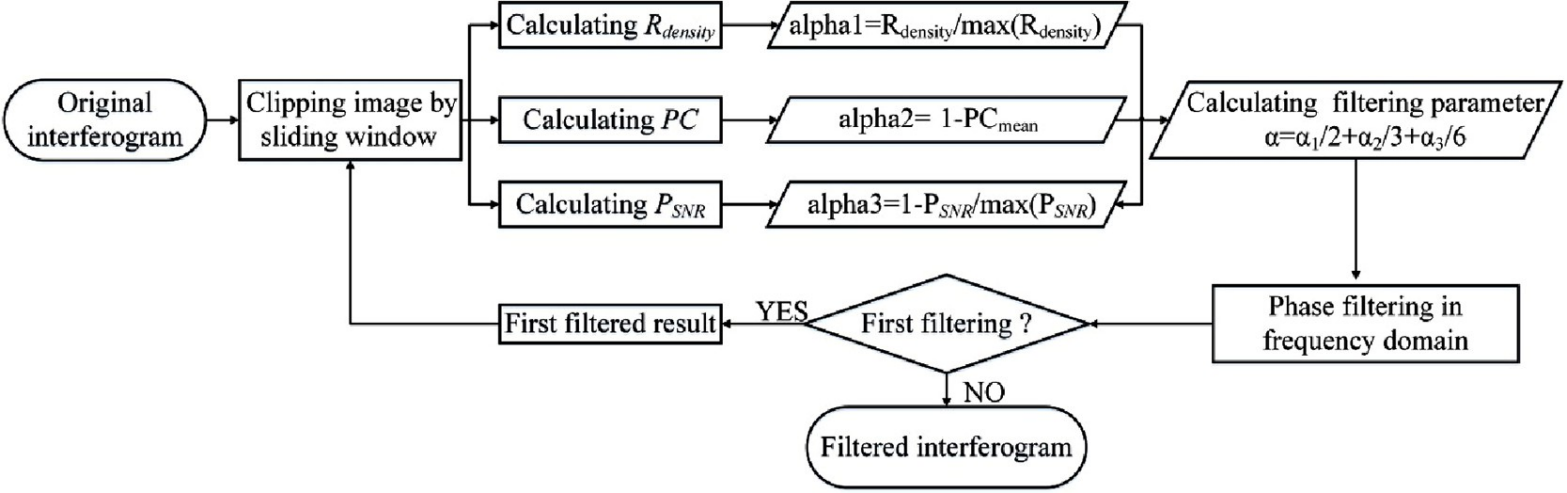
Filtering algorithm flow chart.

The flow chart of the new algorithm is shown in Fig 5, the key is calculating the filter parameters by the multiple quality-guided graphs.

## Results and discussion

Different data were used to verify the effectiveness of the algorithm proposed in this paper. Firstly, the simulated interferometric phase with different level noise were used to verify the effectiveness. The filtering results were compared with Goldstein filter and its improved filtering algorithms. The algorithm was also validated by using TSX/TDX data and real airborne InIRA data.

After filtering, the quantitative indexes were calculated to evaluate the results. The main evaluation indexes include: the residues [34], SPD (sum of phase derivative) [36], PSD (phase standard deviation) [24], CC (correlation coefficient between filtering results and differential phase) [37], EPI (edge preservation index) [38], RMSE (root mean square error) [24]. Generally, the smaller the value of these indicators, the better the filtering effect, except for EPI.

### Simulated data

#### Results of simulated data

In this paper, according to the working mechanism and imaging characteristics of imaging radar altimeter, the interferometric phase was directly simulated, and different degrees of noise were added to the near and far range of the interferogram. The pixels size of the simulated data was 1024*1024, and the noise intensity increases with the increase of the incidence angle. The noise level at the near and far range was -18 dBw and -11.6 dBw respectively.

**Fig 6:**
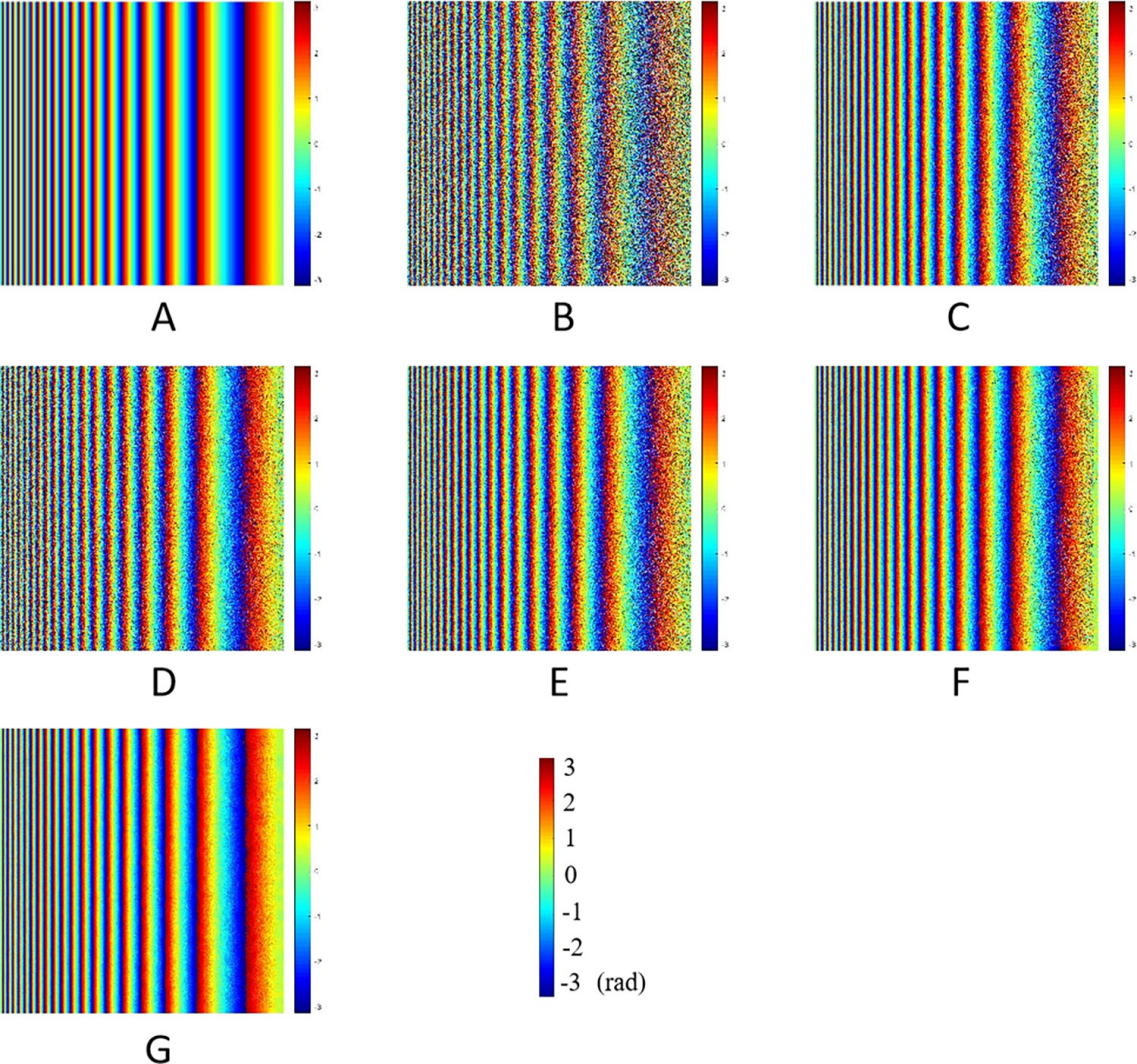
The simulated phases and its filtered results.

The simulated interferometric phase and filtering results of different algorithms were shown in Fig 6. The vertical and horizontal direction represent the azimuth and range direction of interferogram respectively. The interferometric phase without noise and with noise were shown in Fig 6A and 6B respectively.The filtering parameter α of Goldstein was set as 0.5 to avoid over-filtering, and its result was shown in Fig 6C. The improved algorithms were only based on *R*_*density*_, *PC* and *P*_*SNR*_, as shown in Fig 6D–6F, respectively. Among them, the filtering effect of Goldstein algorithm was lower than that of other three improved algorithms. Moreover, the improved algorithm based on *P*_*SNR*_ has better filtering effect. The traditional filtering algorithms can effectively filter the noise in the near range of the interferogram, but there were different degrees of residues in the far range. Compared with others, the filtering effect of the method proposed in this paper was the best, as shown in Fig 6G, which was the closest to the noiseless phase.

#### Discussion of simulated data

The quantitative evaluation indexes of the filtered results of simulated interferometric phase were shown in Table 1. According to the data in the Table 1, the filtering results of the algorithm proposed in this paper were particularly obvious to remove the residues. Although other algorithms had effectively removed some residues, there were still some residual noises. The advantage of the simulated data was that it had the true value of the interferometric phase. From the RMSE, the filtering effect of the new algorithm was the best, and its RMSE was 0.54, followed by the improved Goldstein filtering algorithm based on *P*_*SNR*_, and its RMSE was 1.18. The RMSE of other algorithms was basically similar, which were around 1.5. The CC and EPI can be obtained from original and filtered interferogram. From the specific statistical values, the filtering result of the new algorithm was superior to others, with the values of CC and EPI be 0.32 and 0.79 respectively. The SPD and PSD can be calculated by using the filtered interferogram itself. The filtering effect of new algorithm was the best, with the SPD and PSD of 2.63 × 10^5^ and 0.28 respectively.

**Table 1:** Quantitative evaluation indexes of filtered results of simulated phases.

	Residues	SPD	PSD (3*3)^a^	CC	RMSE	EPI
Original interferogram	139509	/	/	/	/	/
Goldstein filter	24476	10.43e+05	0.95	0.45	1.51	0.58
Filter based on *R*_*density*_	8347	12.18 e+05	1.09	0.41	1.65	0.68
Filter based on *PC*	12564	11.48e+05	1.04	0.43	1.59	0.64
Filter based on *P*_*SNR*_	7371	7.75e+05	0.72	0.51	1.18	0.63
New filter algorithm	10	2.63e+05	0.28	0.32	0.54	0.79

^a^The PSD was calculated using a 3*3 estimation window after filtered.

**Fig 7:**
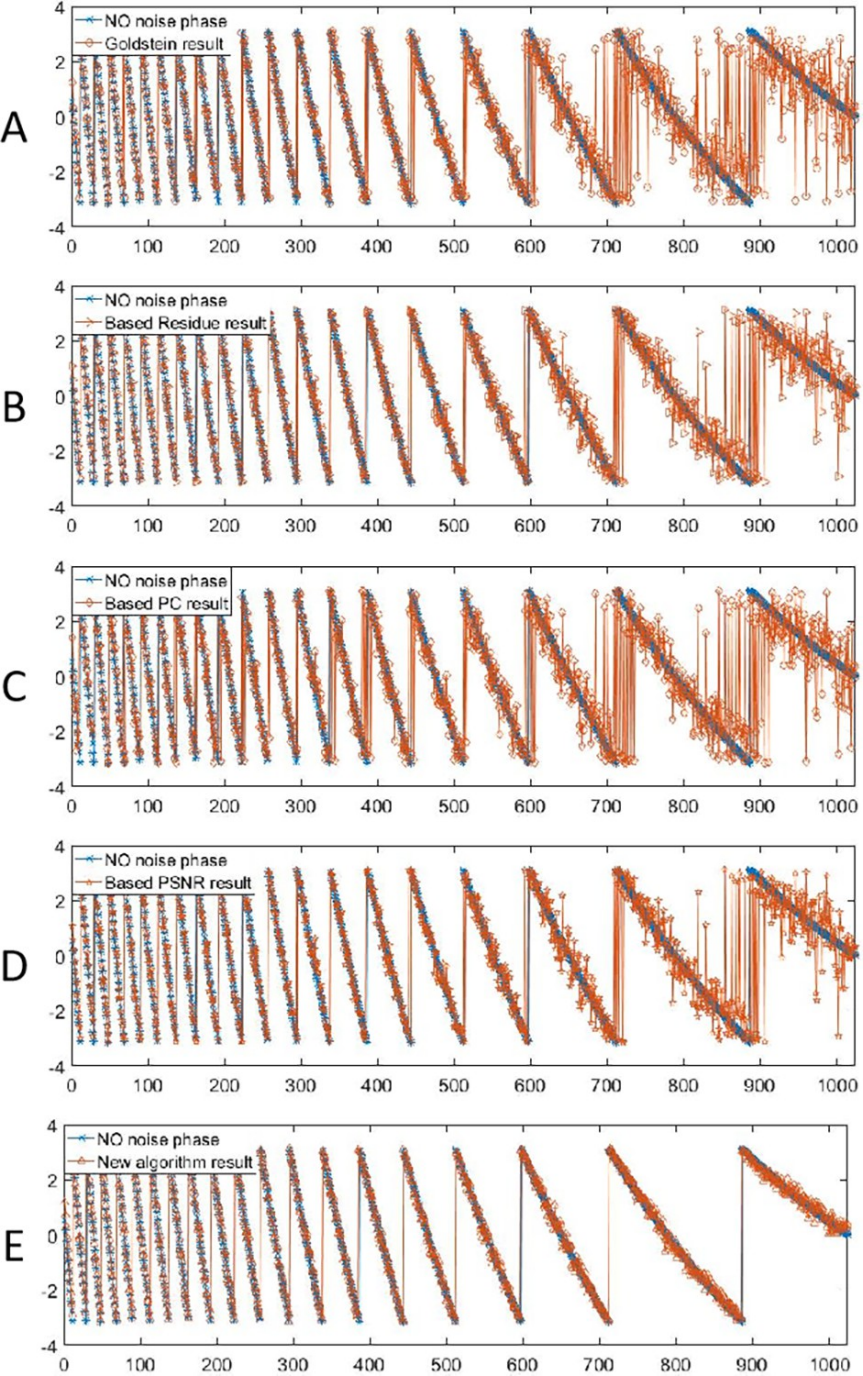
The sections of different filtering results of simulated data.

The sections of Goldstein and its improved filtering results based on *R*_*density*_, *PC* and *P*_*SNR*_ were shown in Fig 7A–7D respectively.The row 20 of the interferogram was selected to make a profile line to obtain the image section of filtering results from the near (left) to far (right) range. The blue curve represents the phase section results of the noiseless interferogram, and the orange curve represents the phase section results after filtering.

Overall, all the filtering algorithms can effectively filter the noise at the near range of the interferogram, so that the phase section lines can be well matched before and after filtering. However, at the far range of the interferogram where the noise was concentrated, the phase section lines of different filtering algorithms had different characteristics. Compared with the noiseless phase, the phase section acquired by the improved Goldstein filtering algorithm based on *P*_*SNR*_ was like that of pure phase, as shown in Fig 7D, but there was a wave or burr at the right part. The phase sections obtained by Goldstein and other improved algorithms have more fluctuations in the right part. As shown in Fig 7E, the filtering result of the new algorithm was well matched with the noiseless phase, both in terms of the whole and the details of the phase transition.

Comprehensively, the proposed algorithm can effectively remove the phase residues not only at the near but also at the far range of the interferogram. It can filter out the phase noise well while maintaining the edge characteristics of the interferometric fringe, to ensure that the filtering result was highly consistent with the noiseless interferometric phase.

### TSX/TDX interferogram

#### Results of the TSX/TDX interferogram

A TSX/TDX interferogram was used to verify the effectiveness of the new algorithm of dual antenna radar interferogram. The TSX/TDX are based on dual station flight mode to obtain image data. The operating parameters of the two radar satellites are basically the same. The wavelength of the two radar satellites is 3.1 cm (X-band). The data product level is SSC (Single look Slant range Complex), the pixel size in range and azimuth direction are about 1.364 m and 2.007 m respectively, and the radar incidence angle is about 40.54°. The specific characteristics of the interferometric pair were shown in Table 2.

**Table 2:** Characteristic of the interferometric pair of TSX/TDX data.

Satellite	Imaging date	Time	Track	Orbit	Product level	Temporal baseline	Spatial baseline /m
TerraSAR-X	2013-01-20	09:46:52	5	31067	SSC	0	275.561
TanDEM-X	2013-01-20	09:46:52	5	31067	SSC		

**Fig 8:**
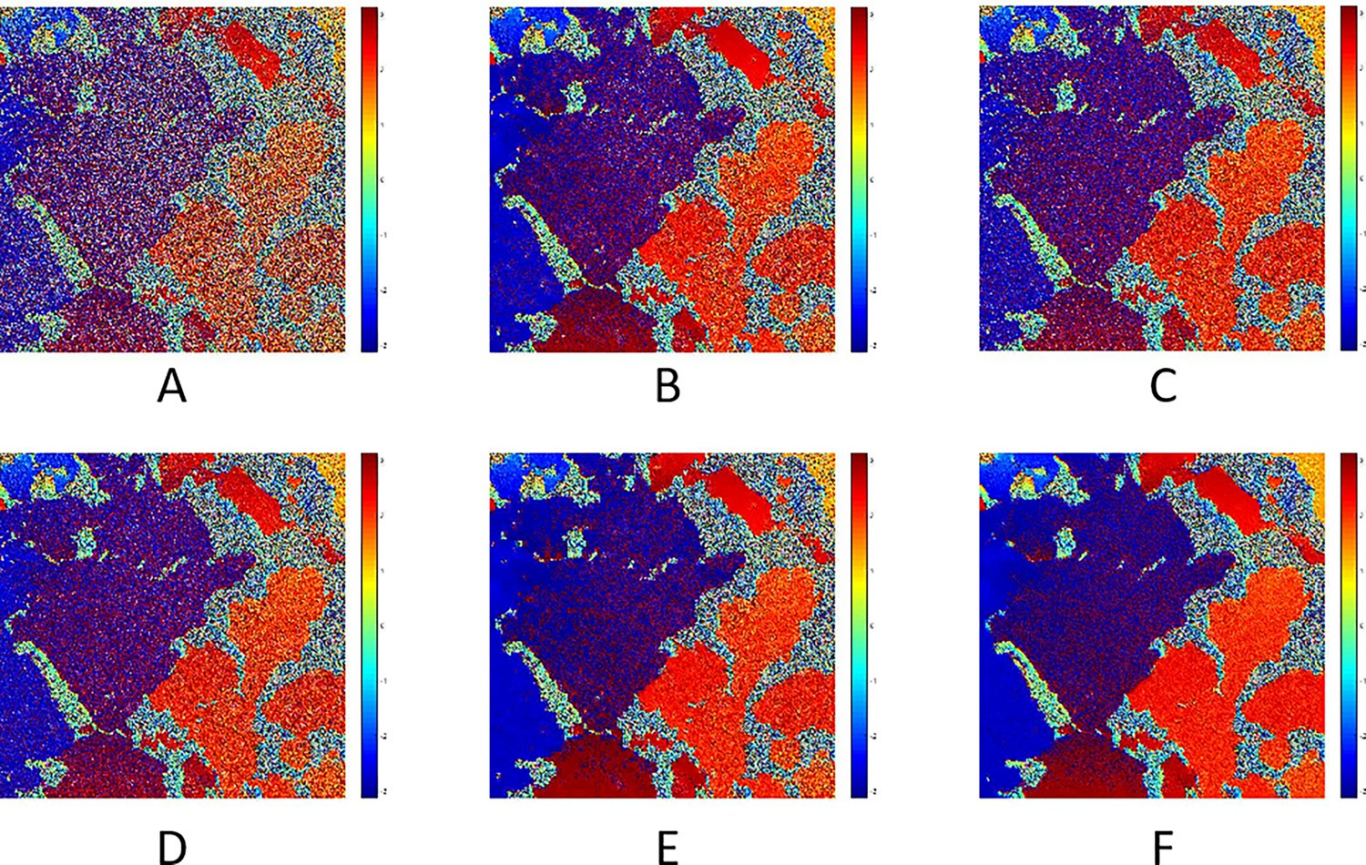
The TSX/TDX interferogram and its filtered results.

The TSX/TDX interferogram and its filtered results of different algorithms were shown in Fig 8. Fig 8A is the original interferogram with an image size of 2000*2000. The actual features include sea ice, leads and seawater. Different features of ground objects can be distinguished from the interferometric phase. Due to the low coherence of seawater, the noise in the interferogram was mainly concentrated in the area where the seawater was distributed.

The results of Goldstein filter and its improved algorithms based on *R*_*density*_, *PC* and *P*_*SNR*_ were shown in Fig 8B–8E respectively. From the perspective of the edge features of sea ice phase (It refers to the interferometric phase where the sea ice was existed) and surface residues, the improved Goldstein filter algorithm based on *P*_*SNR*_ has better noise filtering effect, as shown in Fig 8E, and the edge features of sea ice phase were more obvious, the surface was smoother and more continuous, and the residues was less. But the filtering result of the algorithm proposed in this paper has batter performance, as shown in Fig 8F. It contains less speckle noise, especially on the right side of the image. Compared with the filtering results of other algorithms, its filtering effect was the best.

#### Discussion of the TSX/TDX interferogram

The quantitative evaluation indexes of the results of TSX/TDX interferometric phase were shown in Table 3.

**Table 3:** Quantitative evaluation indexes of filtered results of TSX/TDX data.

	Residues	SPD	PSD (3*3)^a^	CC	EPI
Original interferogram	664760	/	/	/	/
Goldstein filter	256344	6.74e+06	1.65	0.49	0.81
Filter based on *R*_*density*_	238268	7.22e+06	1.76	0.45	0.87
Filter based on *PC*	241197	7.36e+06	1.79	0.45	0.89
Filter based on *P*_*SNR*_	220772	5.44e+06	1.35	0.58	0.76
New filter algorithm	93856	1.91e+06	0.47	0.40	0.74

^a^The PSD was calculated using a 3*3 estimation window after filtered.

In view of the residues, the filtering result of the new algorithm had fewer residues, and the residues’ filtering rate was about 86%. In terms of specific statistics, the CC and EPI of Goldstein filter and its improved algorithms were well relatively, but the filtering results of these algorithms contain the most phase residues. The filtering result of the new algorithm was better than other algorithms in terms of phase residues, SPD and PSD.

Based on the above analysis, the new filtering algorithm can effectively remove noise from the interferogram of the TSX/TDX sea ice data. It can effectively remove the phase residues on the sea ice surface while ensuring the characteristics of the sea ice edge.

### Airborne InIRA data

#### Results of the airborne InIRA data

Next, the experimental data acquired from an airborne InIRA were used. The size of the entire airborne interferogram is 4000*1200 (azimuth*range direction) S1 Fig. In this paper, a 1200*1200 interferogram was obtained by clipping, so that the experimental data includes the image of the whole range direction.

**Fig 9:**
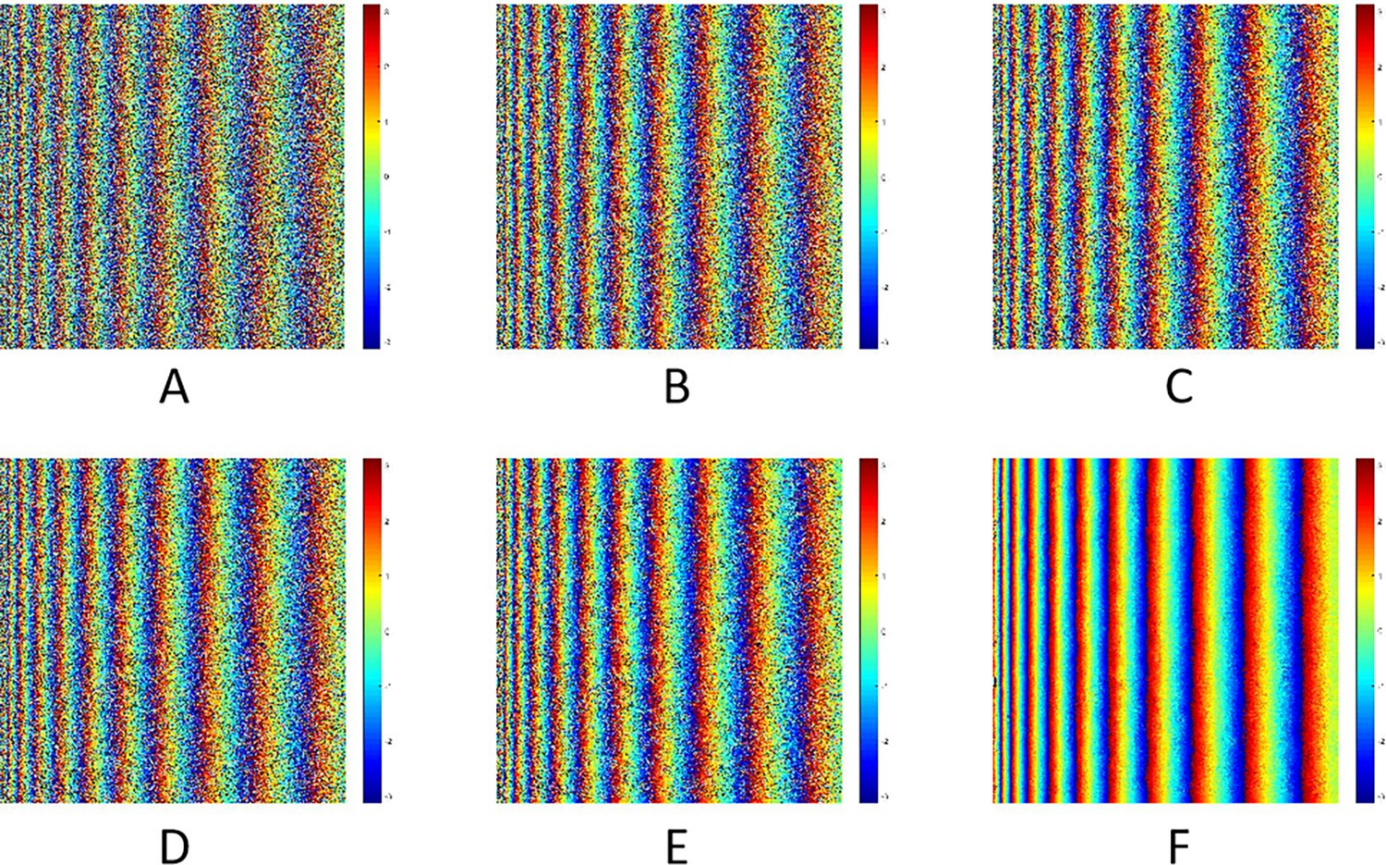
The airborne InIRA data and its filtered results.

The interferometric phase of airborne InIRA and its filtering results of different algorithms were shown in Fig 9. The vertical and horizontal directions represent the azimuth and range directions of the interferogram respectively. Among them, Fig 9A was the original interferogram with a size of 1200*1200, whose interferometric fringe density was sparse from the near range to the far range gradually. The results of Goldstein filter and its improved algorithms based on *R*_*density*_, *PC* and *P*_*SNR*_ were shown in Fig 9B–9E respectively. The filtering result of the new algorithm proposed in this paper was shown in Fig 9F. From the continuity of interferometric fringes and fidelity effect of edge features, the new algorithm was more effective in noise filtering and had the best overall filtering effect, which was helpful for visual interpretation of remote sensing data.

#### Discussion of the airborne InIRA data

The quantitative evaluation indexes of the filtered results of airborne data were shown in Table 4.

**Table 4:** Quantitative evaluation indexes of filtered results of airborne data.

	Residues	SPD	PSD (3*3)^a^	CC	EPI
Original interferogram	169260	/	/	/	/
Goldstein filter	126928	2.27e+06	1.44	0.33	0.89
Filter based on *R*_*density*_	96513	2.11e+06	1.34	0.39	0.83
Filter based on *PC*	119191	2.26e+06	1.43	0.34	0.89
Filter based on *P*_*SNR*_	74085	1.94e+06	1.24	0.44	0.76
New filter algorithm	174	0.52e+06	0.35	0.39	0.82

^a^The PSD was calculated using a 3*3 estimation window after filtered.

According to the statistical results in the Table 4, the filtering result of the new algorithm proposed in this paper was better than others on the whole. The residues’ filtering rate was close to 100%, and the values of CC and EPI were 0.39 and 0.82, the SPD and PSD were 0.52 × 10^6^ and 0.35, respectively.

Comprehensively, in terms of the airborne data of InIRA, the filtering algorithm proposed in this paper that considering the multiple quality-guided graphs, which can not only in the near range of interferogram but also in the far range was able to filter out noise. Moreover, it can ensure the interferometric fringe edge features. Both from subjective visual and comprehensive evaluation indexes, the filtering algorithm proposed in this paper has great filtering results.

## Conclusions

Aiming at the phase filtering of InIRA data, an adaptive filtering algorithm combined with multiple quality-guided graphs was proposed. The results showed that: (1) The algorithm proposed in this paper can effectively filter the noise while ensuring the edge characteristics of the interferometric fringe. The section of filtering result can well match with the section of simulated pure interfeometric phase. (2) It can effectively remove the noise from the interferogram of TSX/TDX sea ice data with the residues filtering of 86%. The phase residues on the sea ice surface were effectively removed, and the identification degree between sea ice and water was improved. The algorithm proposed in this paper that considering the multiple quality-guided graphs, can better reflect the noise characteristics from near and far range of interferogram. In some extent, it can effectively deal with the interferometric noise pollution in InIRA. It provided a method for the data processing and application of InIRA in the future. However, in the case of the lack of true value (SWOT and Chinese Tiangong-2 data) as a reference, the selection of the multiple quality-guided graphs lacks certain objective judgment criteria. Finally, as a future work, we plan to select the quality-guided graphs according to SWOT or Tiangong-2 data. At the same time, we will optimize the distribution of weights of the filter parameter components.

## Supporting information

S1 Fig The entire interferogram of airborne InIRA. (TIF)
