# Supplementary figures and images for: A modified Goldstein filter for interferogram denoising of interferometric imaging radar altimeter based on multiple quality-guided graphs
Source: PLoS One. 2024 Aug 8;19(8):e0308636. doi: 10.1371/journal.pone.0308636 (PMC11309471; doi:10.1371/journal.pone.0308636)

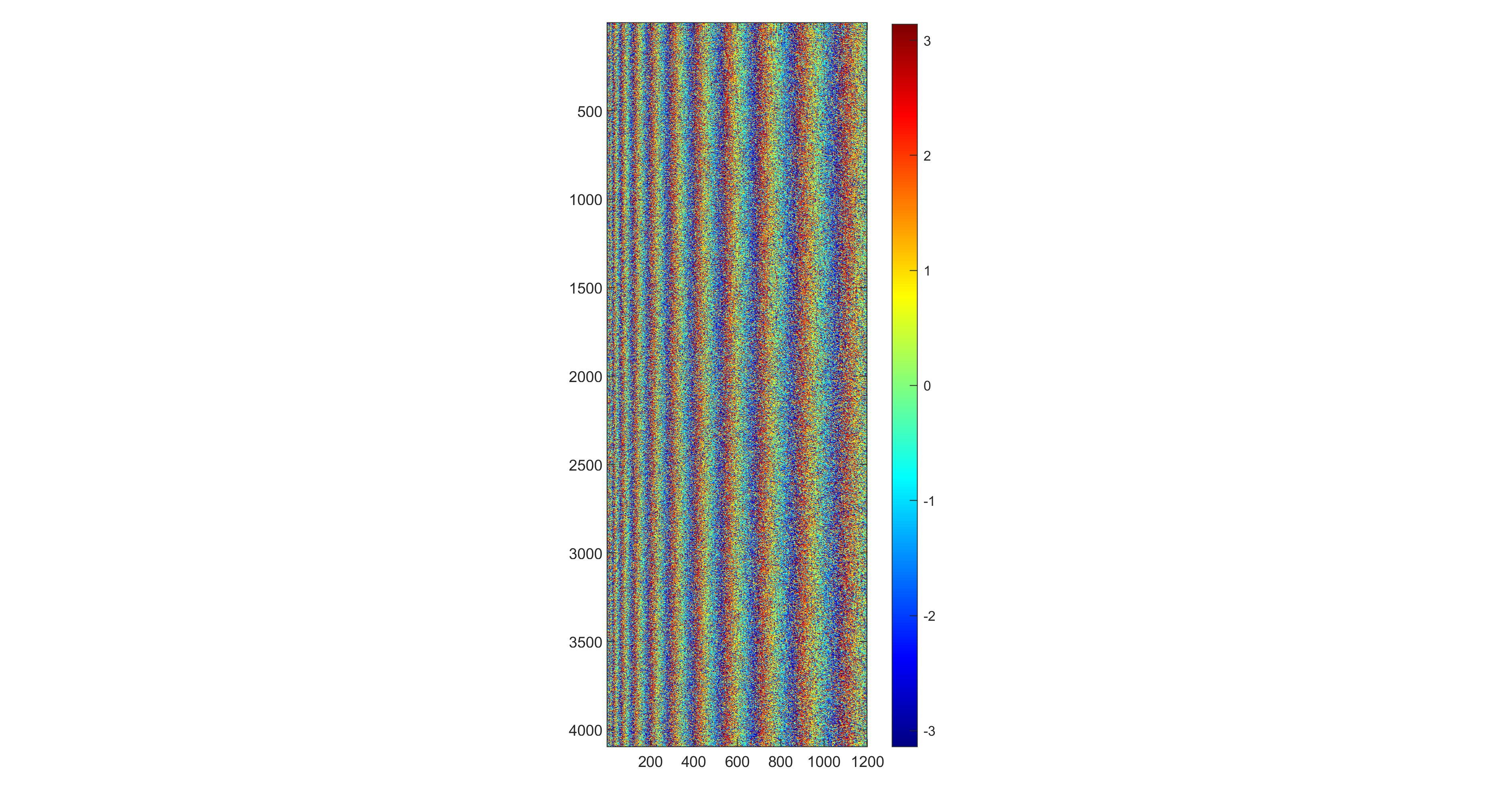

Supplement: S1 Fig — (TIF) [file pone.0308636.s001.tif]
